# KLK12 Regulates MMP-1 and MMP-9 via Bradykinin Receptors: Biomarkers for Differentiating Latent and Active Bovine Tuberculosis

**DOI:** 10.3390/ijms232012257

**Published:** 2022-10-14

**Authors:** Yuanzhi Wang, Mengjin Qu, Yiduo Liu, Haoran Wang, Yuhui Dong, Xiangmei Zhou

**Affiliations:** College of Veterinary Medicine, China Agricultural University, Beijing 100193, China

**Keywords:** *Mycobacterium bovis*, KLK12, latent tuberculosis infection, diagnosis

## Abstract

It has been established that kallikrein12 (KLK12) expression is closely related to bovine tuberculosis (bTB) development. Herein, we sought to clarify the regulatory mechanism of KLK12 and its application in tuberculosis diagnosis. KLK12 knockdown macrophages were produced by siRNA transfection. Bradykinin receptors (BR, including B1R and B2R) were blocked with specific inhibitors. Mannose-capped lipoarabinomannan (ManLAM) was extracted from *Mycobacterium bovis* (*M. bovis*) and used to study the mechanism of KLK12 activation. In addition, we constructed different mouse models representing the latent and active stages of *M. bovis* infection. Mouse models and clinical serum samples were used to assess the diagnostic value of biomarkers. Through the above methods, we confirmed that KLK12 regulates MMP-1 and MMP-9 via BR. KLK12 upregulation is mediated by the *M. bovis*-specific antigen ManLAM. KLK12, MMP-1, and MMP-9 harbor significant value as serological markers for differentiating between latent and active bTB, especially KLK12. In conclusion, we identified a novel signaling pathway, KLK12/BR/ERK/MMPs, in *M. bovis*-infected macrophages, which is activated by ManLAM. From this signaling pathway, KLK12 can be used as a serological marker to differentiate between latent and active bTB. Importantly, KLK12 also has enormous potential for the clinical diagnosis of human tuberculosis (TB).

## 1. Introduction

*Mycobacterium bovis* (*M. bovis*) is a member of the *Mycobacterium tuberculosis* complex (MTC), widely acknowledged as the etiology of bovine tuberculosis. *M. bovis* is distributed worldwide, causing substantial economic losses to farm communities [1]. After *Mycobacterium tuberculosis* (*Mtb*), *M. bovis* is widely acknowledged as the most common etiological agent of TB, accounting for about 5% of the global tuberculosis burden [2,3]. In this regard, people who utilize unpasteurized milk or have direct contact with infected animals are more susceptible to *M. bovis* infection [2]. It has been established that TB caused by either *Mtb* or *M. bovis* exhibits the same clinical symptoms and similar radiological and histopathological patterns [2]. *M. bovis* mainly affects the respiratory system of the host, leading to typical granulomatous lesions in pulmonary tissues with visible areas of necrotic cores surrounded by epithelioid macrophages and lymphocytes. Macrophages represent the first line of defense against *Mtb* and *M. bovis* infection and play an active role in the modulation of the early immune response [4]. Most importantly, the immune response limits the spread of *Mtb* and walls off the bacteria in dense cellular masses known as granulomas or tubercular lesions [5,6]. Although the innate immune response is an essential determinant of the disease, the possible outcome of infection varies among susceptible individuals, and the factors involved remain largely unknown [7,8].

Kallikrein-related peptidases (KLKs) belong to a family of fifteen closely related serine proteases with either trypsin- or chymotrypsin-like activity. KLKs are well-known to activate the signaling of both bradykinin receptors (B1R and B2R) and protease-activated receptors (PARs), participating in physiological processes and various pathophysiological conditions [9,10]. An increasing body of evidence from recently published studies suggests that KLKs participate in various infectious and degenerative diseases [11,12,13]. At least 11 KLKs, including KLK-1, 3, and 5-14, have been reported to be secreted in healthy or diseased lungs. KLKs are more commonly observed in the bronchioles than in the alveoli [14], suggesting that KLKs are an important component of bronchial secretions and participate in the pulmonary response to external insults. Ample evidence substantiates that KLK12 mRNA levels are upregulated in cancer tissues, including gastric, breast, prostate, and colorectal cancer [15,16]. It is widely thought that KLK12 has significant value as a tumor biomarker, given its involvement in tumorigenesis [15]. However, the role of KLK12 in TB remains unclear.

Matrix metallopeptidases (MMPs) are related to the family of proteolytic enzymes capable of degrading extracellular matrix (ECM) components and may act as inflammatory mediators. Over the years, the bradykinin-mediated secretion of MMPs has been extensively explored in various tissues [17,18,19]. It has been reported that B1R agonist treatment increases the proliferation of estrogen-sensitive breast cancer cells. Nonetheless, B1R signaling is also involved in the upregulation of MMP-2 and MMP-9 through an ERK-dependent pathway [20]. Similarly, B2R has been shown to regulate MMP-9 secretion via ERK-1/-2 signaling in trabecular meshwork cells [21]. Importantly, MMPs can regulate the expression and function of various KLKs, suggesting a potential interaction between KLKs and MMPs in physiological and pathological conditions [22]. It is widely believed that KLK-mediated B1R and B2R signaling are involved in MMP secretion in TB, but no study has hitherto been reported [23]. In the present study, we found that *M. bovis* infection upregulated the expression of KLK12, B1R/B2R, and MMP-1/-9 in murine macrophages and mice. Interestingly, the knockdown of KLK12 significantly downregulated the expression of B1R/B2R and MMP-1/-9, whereas the inhibition of B1R/B2R only downregulated the levels of MMP-1/-9. Thus, the signaling pathway of KLK12 through bradykinin receptors regulating MMP-1/-9 was revealed in *M. bovis*-infected murine macrophages. Importantly, the potential interaction between KLKs and MMPs has been associated with the progression of bTB. B1R and B2R can be developed as effective therapeutic targets for adjuvant TB treatment as a bridge between them. Moreover, these biomarkers can increase the sensitivity of the diagnosis of *M. bovis* infection and distinguish between active bTB and latent tuberculosis infection (LTBI) or between LTBI and *M. bovis*-uninfected healthy individuals. Our findings may help improve the diagnostic efficiency and elimination of bTB in high-burden countries.

Since this article uses a large number of acronyms, we have included the list ‘Acronyms’ at the end for the convenience of the reader.

## 2. Results

### 2.1. KLK12 Mediates MMP-1 and MMP-9 Expression

**Figure 1:**
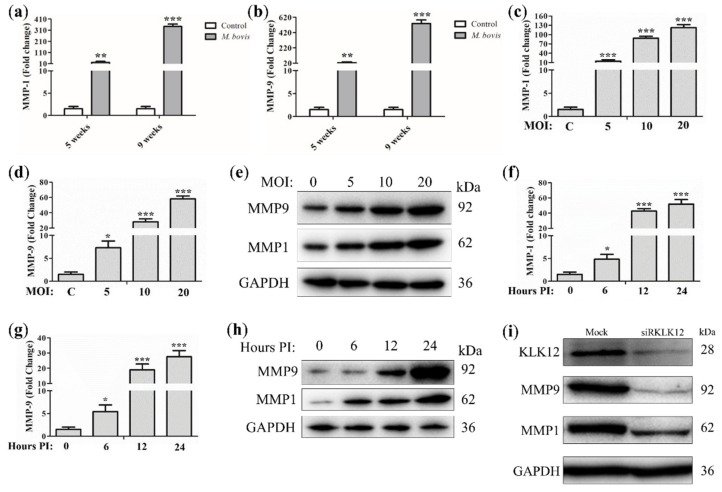
Knockdown of KLK12 downregulates expression of MMP-1 and MMP-9 in *M. bovis*-infected RAW264.7. Mice (*n* = 10) were intranasally infected with *M. bovis* C68004 (at a dose of 200 CFU/mouse) or with PBS. Mice were sacrificed after 5 and 9 weeks of infection, and lung tissues were collected. (**a**) MMP-1 and (**b**) MMP-9 expression levels were determined by Quantitative Real-Time PCR (qRT-PCR). RAW264.7 cells were infected with *M. bovis* C68004 at variable MOIs (5, 10, and 20) and incubated for 24 h. MMP-1 and MMP-9 expression levels were determined by (**c**,**d**) qRT-PCR and (**e**) Western blot. Next, RAW264.7 cells were infected with *M. bovis* C68004 at an MOI of 10 and incubated for indicated periods, i.e., 0, 6, 12, and 24 h. MMP-1 and MMP-9 expression levels were determined by (**f**,**g**) qRT-PCR and (**h**) Western blot. KLK12 was then knocked down in *M. bovis*-infected RAW264.7 cells by transfection with siRNA, and the altered expression levels of (**i**) MMP-1 and MMP-9 after KLK12 knockdown were detected by Western blot. The data shown in the bar graph represent the mean ± SD of three independent in vitro experiments (* *p* < 0.05; ** *p* < 0.01; *** *p* < 0.001).

We investigated the in vivo and in vitro secretion of MMP-1/-9 in *M. bovis* infection. During the in vivo experiment, C57BL/6 mice were challenged with *M. bovis* C68004. At weeks 5 and 9 of infection, a significant increase in MMP-1 (Figure 1a) and MMP-9 (Figure 1b) expression was observed in the lungs of infected mice compared to uninfected control mice. During the in vitro study, we infected murine RAW264.7 cells with *M. bovis* at the indicated multiplicity of infection (MOI) and incubated them for the specified periods. We found that the transcription (Figure 1c,d) and protein levels (Figure 1e) of MMP-1 and MMP-9 were dose-dependently upregulated in *M. bovis*-infected RAW264.7 cells. Next, we infected RAW264.7 cells at an MOI of 10 and incubated them for the indicated periods, i.e., 0, 6, 12, and 24 h. The transcription (Figure 1f,g) and protein levels (Figure 1h) of MMP-1 and MMP-9 in RAW264.7 cells were enhanced in a time-dependent manner. These findings suggest that MMP-1 and MMP-9 expression may be closely related to the progression of TB disease. Accordingly, we sought to explore the regulatory mechanism between KLK12 and MMP-1/-9 and assess their value for the differential diagnosis between bTB and TB. In the next experiment, we explored the effect of KLK12 knockdown on the expression levels of MMP-1 and MMP-9. A significant reduction in the expression of MMP-1 and MMP-9 was observed in KLK12 knockdown RAW264.7 cells at 24h post-infection with *M. bovis* compared to control cells (Figure 1i). Our results suggest that KLK12 could play a role in the regulation of MMP-1 and MMP-9 expression in *M. bovis* infection. However, the underlying regulatory mechanisms remain unclear, highlighting the need for further investigation.

### 2.2. KLK12 Mediates MMP-1 and MMP-9 Expression via BR/ERK Signaling

**Figure 2:**
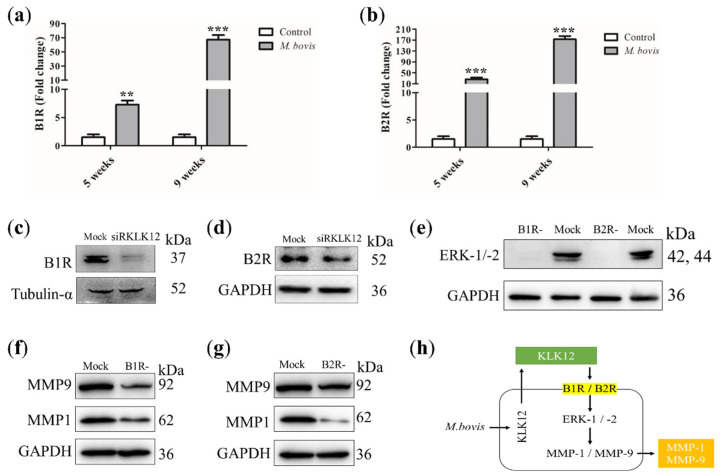
Blocking BR downregulates ERK and MMP-1/-9 expression. Mice (*n* = 10) were intranasally infected with *M. bovis* (at a dose of 200 CFU/mice) or with PBS. Mice were sacrificed after 5 and 9 weeks of infection, and lung tissues were collected. (**a**) B1R and (**b**) B2R expression was determined by Quantitative Real-Time PCR (qRT-PCR). RAW264.7 cells were transfected with siRKLK12 and negative control siRNA (20uM). After 48 h of transfection, the cells were infected with *M. bovis*. After 24 h, samples were collected. Then, (**c**) B1R and (**d**) B2R expression was determined by Western Blot analysis. To validate our hypothesis, RAW264.7 cells were processed to block bradykinin receptors and infected with *M. bovis*. After 24 h, samples were collected. (**e**) ERK-1/-2 and (**f**,**g**) MMP-1/-9 expression was determined by Western Blot analysis. (**h**) The regulation of MMP-1/-9 expression by *M. bovis* through the BR/ERK signaling pathway is represented schematically. Data represent the mean ± SD of three independent experiments (** *p* < 0.01; *** *p* < 0.001).

Both B1R and B2R are known to activate ERK-1/-2 kinases, ultimately leading to the increased expression of MMP-1 and MMP-9 [23]. Therefore, we hypothesized that KLK12 regulates MMP-1 and MMP-9 expression via BR. Accordingly, we investigated whether *M. bovis* infection could stimulate the expression of bradykinin receptors, B1R and B2R, in vivo and in vitro. High expression of B1R (Figure 2a) and B2R (Figure 2b) was observed in the lungs of infected mice compared to uninfected control mice. Next, the expression of B1R and B2R in RAW264.7 cells with KLK12 knockdown was examined. In fact, as we hypothesized, B1R (Figure 2c) and B2R (Figure 2d) expression levels were significantly downregulated in KLK12 knockdown RAW264.7 cells at the indicated time points of *M. bovis* infection compared to control cells. These results suggest that KLK12 can regulate BR signaling. To assess whether KLK12 mediates MMP-1 and MMP-9 through BR/ERK signaling, B1R and B2R were blocked with specific inhibitors. RAW264.7 cells were pretreated with inhibitors for 30 min and then infected with *M. bovis*. After 24 h, the expression levels of ERK-1/-2, MMP-1, and MMP-9 were detected. Western blot results showed that the inhibition of either B1R or B2R could downregulate the expression of ERK-1/-2 (Figure 2e) and MMP-1/-9 (Figure 2f,g), corroborating that the KLK12-mediated regulation of MMP-1 and MMP-9 expression involves BR/ERK pathways (Figure 2h). In summary, we identified a novel signaling pathway, KLK12/BR/ERK/MMPs, in *M. bovis*-infected macrophages. Previous studies have demonstrated that MMP-1 and MMP-9 can drive the immunopathology of human TB and have been used as therapeutic targets and diagnostic markers. Therefore, KLK12 should have a similar potential as an upstream signal for MMP-1/-9, but its specificity and sensitivity remain to be evaluated.

### 2.3. KLK12 Upregulation Is Mediated by the M. bovis-Specific Antigen ManLAM

**Figure 3:**
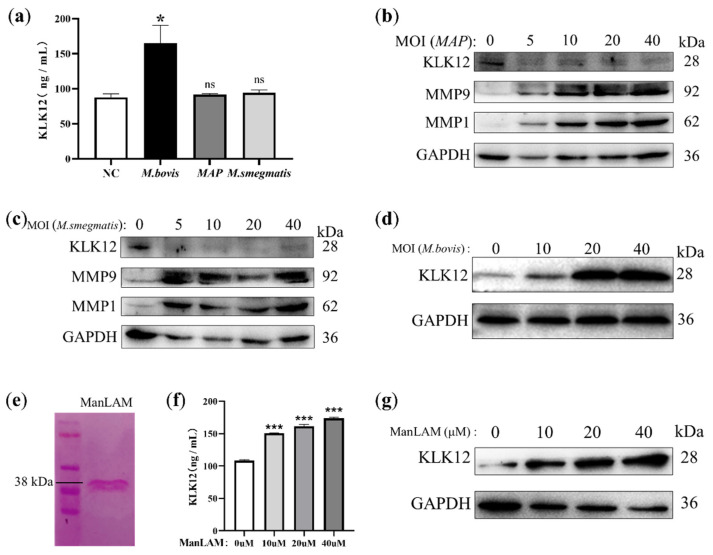
Differences in upregulation of KLK12 secretion and expression by *MAP*, *M. smegmatis*, and *M. bovis* are due to ManLAM. RAW264.7 cells were infected with *MAP*, *M. smegmatis*, or *M. bovis*, and (**a**) KLK12 concentration in cell culture supernatant was measured by ELISA kit 24 h after infection. Western blot analysis to detect KLK12 or MMP-1/-9 expression in cells infected with (**b**) *MAP*, (**c**) *M. smegmatis*, and (**d**) *M. bovis* at various MOIs. (**e**) In this study, ManLAM was extracted from the cell wall of *M. bovis* and subjected to SDS-PAGE electrophoresis, followed by glycogen PAS staining of the isolated gel. Using various concentrations of ManLAM on RAW264.7 cells, KLK12 was detected in cell culture supernatants and cells using (**f**) ELISA and (**g**) Western blot, respectively. Data represent the mean ± SD of three independent experiments (* *p* < 0.05; *** *p* < 0.001).

In this study, we infected RAW264.7 cells with *M. bovis* C68004 and two non-tuberculous mycobacteria species (NTMs) (*Mycobacterium paratuberculosis* (*MAP*) and *Mycobacterium smegmatis* (*M. smegmatis*)) separately, and the KLK12 concentration in the cell culture supernatant was measured 24 h after infection. The results showed that only *M. bovis* infection increased KLK12 secretion compared to uninfected cells, whereas *MAP* and *M. smegmatis* did not have this effect (Figure 3a). We then examined the expression levels of KLK12 and MMP-1/-9 in RAW264.7 cells infected with NTMs and observed that *MAP* (Figure 3b) and *M. smegmatis* (Figure 3c) infection only upregulated MMP-1/-9 but downregulated KLK12. However, *M. bovis* can upregulate the expression of both KLK12 (Figure 3d) and MMP-1/-9 (Figure 1e). These results suggest that, in addition to the previously reported mechanism, in which Mycobacterium is recognized by Toll-like receptors-1/-2 (TLR-1/-2) [24] and thus activates downstream signaling pathways leading to the upregulation of MMP-1/-9 expression, there are differences between *M. bovis* and NTMs in the activation of the KLK12/BR/ERK/MMPs signaling pathway. This may be due to differences in certain components of the cell walls of *M. bovis* and NTMs, as the innate immune response to Mycobacterium is initiated after its unusual lipid-rich cell wall is recognized. Lipoarabinomannan (LAM), a Mycobacterium cell wall lipoglycan, is a major virulence factor with the ability to manipulate the host immune system. The presence and structure of capping allow the classification of LAM molecules into ManLAM, PILAM, and AraLAM classes. Among them, ManLAM was only found in pathogenic species, including *M. bovis*, *Mtb*, and *Mycobacterium leprae* [25]. Although both ManLAM and PILAM belong to LAM molecules, their roles in the triggering of certain signaling pathways are diametrically opposed [26]. Therefore, we extracted ManLAM from the cell wall of *M. bovis* C68004 (Figure 3e) and applied it to RAW264.7 cells. ManLAM also upregulated KLK12 secretion (Figure 3f) and expression (Figure 3g) in a dose-dependent manner, consistent with the results in *M. bovis*-infected macrophages (Figure 3d). These results indicate that KLK12 upregulation is mediated by the *M. bovis*-specific antigen ManLAM. Since ManLAM is not present in NTMs such as *MAP* and *M. smegmatis*, these NTMs can upregulate MMP-1/-9 but do not have the ability to upregulate KLK12.

### 2.4. Establishment of LTBI Model in Mice Infected with Ultra-Low Doses of M. bovis

**Figure 4:**
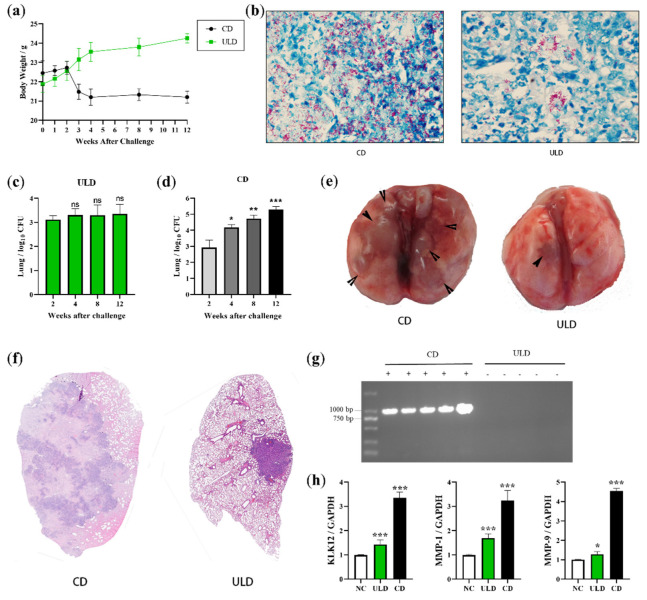
Establishment of LTBI model in B6 mice infected with ULD of *M. bovis*. B6 mice (*n* = 20) were intranasally infected with *M. bovis* (at doses of 200 CFU/mice or 2 CFU/mice) or with PBS. (**a**) Subsequently, weights were measured at the same time points, and weight change curves were plotted. Three mice were sacrificed after 2, 4, and 8 weeks of infection, organs and sera were collected, (**e**) and gross lung lesions were observed. (**c**,**d**) Then, the inferior lobe of the right lung and the whole spleen were ground and coated on 7H10 agar plates for bacterial load statistics. The left lung was fixed with formaldehyde, and tissue sections were stained with (**f**) H&E and (**b**) acid-fast stains. (**g**) DNA of mice pharyngeal swabs was extracted for PCR detection of *M. bovis*. (**h**) RNA of lung tissues was extracted and reverse transcribed into cDNA for qPCR detection of KLK12, MMP-1, and MMP-9 transcription levels. Analysis of data using GraphPad Prism software (* *p* < 0.05; ** *p* < 0.01; *** *p* < 0.001).

The current estimate is that ~2 billion individuals, making up a quarter of the world’s population, are latently infected with *Mtb*. LTBI can reactivate even decades after infection to cause transmissible disease [27]. Therefore, the rapid and accurate identification of active TB and LTBI for precise treatment is essential to reduce the incidence of TB. Previous studies have identified MMPs as markers of disease severity and bacterial burden and as potential candidates for non-sputum-based biomarkers that distinguish pulmonary TB from LTBI and healthy individuals [28,29]. Therefore, KLK12, as a regulator of MMP-1/-9, should also have diagnostic marker potential. Furthermore, the diagnostic potential of LAM has led it to become the current gold-standard biomarker for point-of-care (POC) TB diagnostic tests [30]. KLK12, as an activation product of ManLAM, further highlights its potential as a diagnostic marker. In order to test our hypothesis, it is necessary to use the appropriate TB model, especially an LTBI model. Interestingly, Courtney R. Plumlee et al. reported that infecting mice at an ultra-low dose (ULD) could be used to develop a TB model that closely resembles the human disease [31]. Based on this study, we attempted to infect C57BL/6 (B6) mice with a ULD of *M. bovis* (2 CFU) to establish an LTBI model. We also infected B6 mice with the conventional dose (CD) of *M. bovis* (200 CFU) and performed a long-term (12 weeks) comparative analysis with ULD-infected mice. It was observed that CD-infected mice showed a trend of weight loss 3 weeks after infection (Figure 4a). However, ULD-infected mice did not show weight loss (Figure 4a), which is a major clinical symptom of active TB. In view of this result, we conducted an in-depth study of lung lesions, bacterial load, and bacterial excretion in two groups of mice. Although *M. bovis* was observed in the lungs of both groups of mice in antacid-stained lung tissue sections (Figure 4b), the bacterial load in the lungs of ULD-infected mice (Figure 4c) was significantly lower than that of CD-infected mice (Figure 4d). We performed serial statistical analyses of lung lesions and bacterial loads in the two groups of mice. The lung CFU in CD-infected mice continued to increase with the duration of infection (Figure 4d), and a large number of raised nodules were visible on the surface of the lungs (Figure 4e). Hematoxylin and eosin (H&E)-stained sections of lung tissue from CD-infected mice at 4 weeks post-infection showed extensive lesions (Figure 4f). Most notably, the presence of *M. bovis* in the pharyngeal swabs of CD-infected mice was detected by PCR (Figure 4g). The above results indicate that mice infected with the CD of *M. bovis* have no latent infection period and belong to the model of active TB. However, ULD-infected mice are better able to simulate the course of LTBI. Firstly, although *M. bovis* was also present in the lungs of ULD-infected mice (Figure 4b), the bacterial load was stable at a low level for a long time (Figure 4c). Secondly, no raised nodules were seen in the lungs of ULD-infected mice, and only one lesion (Figure 4e) was observed as an isolated granuloma on H&E-stained sections (Figure 4f). Finally, ULD-infected mice showed no clinical symptoms of active TB, did not lose weight (Figure 4a), and did not excrete bacteria into the external environment (Figure 4g). Similar to the phenotype in bovine LTBI, *M. bovis* infection persisted in ULD-infected mice without clinical symptoms, exhibiting a slow-replication latent state. These results suggest that ULD-infected mice can simulate bovine LTBI, but the findings for CD-infected mice were more similar to active bTB. Using these two infection models, we confirmed that the transcription levels of KLK12, MMP-1, and MMP-9 in lung tissue were positively correlated with the initial dose of infection (Figure 4h). Taken together, these findings suggest that KLK12- and KLK12-regulated MMP-1 and MMP-9 expression can potentially be used as biomarkers for discriminating between LTBI and active bTB.

### 2.5. KLK12 Could Serve as a Serological Biomarker to Detect bTB Disease States

**Figure 5:**
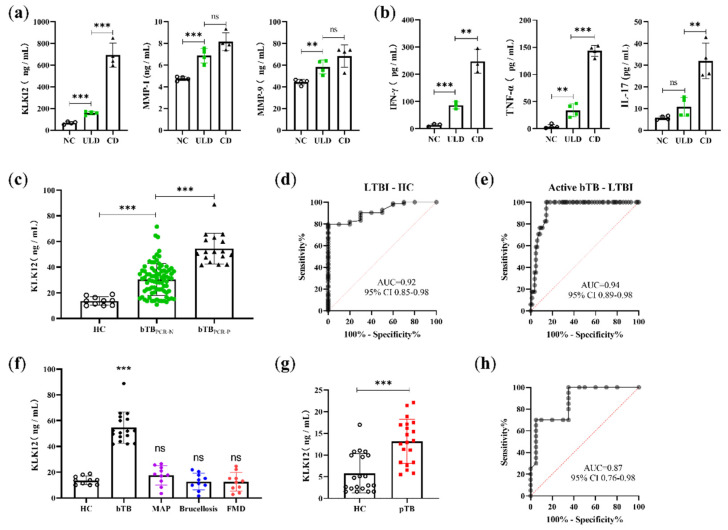
B6 mice (*n* = 30) were intranasally infected with *M. bovis* (at doses of 200 CFU/mice or 2 CFU/mice) or with PBS. Four mice per group were sacrificed after 4 weeks of infection. (**a**) Sera were collected, and KLK12, MMP-1, and MMP-9 levels were determined by ELISA kits. (**b**) The cytokine (IFN-γ, TNF-α, and IL-17) levels in the sera of mice were detected by ELISA kits. (**c**) KLK12 levels in sera of healthy control (HC), bTB PCR-negative (bTB_PCR-N_), and bTB PCR-positive (bTB_PCR-p_) bovine samples were determined using ELISA kits. ROC curves were drawn to evaluate the potential of KLK12 for the diagnosis of (**d**) LTBI and (**e**) active bTB. (**f**) Then, the same ELISA kits were used to detect KLK12 in bovine serum infected with different diseases. (**g**) The ELISA kit was also used to detect KLK12 in the sera of healthy humans (HCs) and active pulmonary TB patients (pTB), and (**h**) an ROC curve was drawn according to the detection results. Analysis of data using GraphPad Prism software (** *p* < 0.01; *** *p* < 0.001).

ELISA kits were used to detect serum levels of KLK12, MMP-1, and MMP-9 in mice with different infection doses, and the results were consistent with findings in lung tissues. Serum KLK12, MMP-1, and MMP-9 were significantly upregulated in mice after *M. bovis* infection, and the degree of upregulation was positively correlated with the initial infection dose. However, only KLK12 exhibited the most significant difference among the negative control (NC), ULD, and CD groups (Figure 5a). Considering that KLK12 is more sensitive (Figure 5a) and specific (Figure 3a) than MMP-1/-9, this result indicates that KLK12 has superior diagnostic value as a serological biomarker of bTB. Several studies have been conducted to apply multiple cytokine microbead arrays to plasma to differentiate between LTBI and active TB. Therefore, we evaluated a number of cytokines using the LTBI and active TB models established in this study. Among them, IFN-γ, TNF-α, and IL-17 also showed their potential as diagnostic biomarkers in discriminating between ULD and CD groups (Figure 5b). However, only IFN-γ exhibited the most significant difference among the NC, ULD, and CD groups (Figure 5b), as did KLK12 (Figure 5a). Subsequently, we sought to explore whether KLK12 exhibits good sensitivity and specificity when applied as a serological biomarker for bTB. We collected a large number of serum samples from cattle farms to assess the diagnostic potential of KLK12. In this experiment, 17 bTB PCR-positive (bTB_PCR-p_), 83 bTB PCR-negative (bTB_PCR-N_), and 10 bTB healthy control (HC) cattle were screened using the tuberculin skin test (TST) and the PCR detection of nasal swab secretion. The venous blood of these cattle was collected aseptically, and the serum KLK12 concentrations were detected by ELISA. As expected, the results were consistent with findings observed in mice; the serum KLK12 levels in bTB_PCR-p_ and bTB_PCR-N_ groups were statistically significantly higher than that in the HC group (Figure 5c). The cutoff values for distinguishing each group were determined by receiver operating characteristic (ROC) analysis (Figure 5d,e). At a cutoff value of 16.49 ng/mL (sensitivity, 81.93%; specificity, 80%) (Table 1), KLK12 could distinguish bovine LTBI from healthy bovine samples (area under the curve (AUC), 0.92; 95% confidence interval (CI), 0.85–0.98) (Figure 5d) with an accuracy of 81.72% (76/93). In addition, at a cutoff value of 41.55 ng/mL (sensitivity, 100%; specificity, 85.54%) (Table 1), KLK12 could distinguish LTBI from active bTB bovine samples (AUC, 0.94; 95% CI, 0.89–0.98) (Figure 5e), yielding an accuracy of 88% (88/100). These results demonstrate that serum KLK12 can effectively differentiate between LTBI and healthy bovine samples and be used as a serological biomarker for discriminating between LTBI and active bTB. Other common clinical diseases of cattle, such as bovine paratuberculosis, brucellosis, and foot and mouth disease (FMD), did not affect the diagnosis of active bTB (Figure 5f). In addition, the clinical diagnostic value of KLK12 was also demonstrated in 20 healthy human controls (HCs) and 20 active pulmonary TB patients (pTB). Serum KLK12 levels in healthy subjects were significantly higher than those in active TB subjects (Figure 5g). At a cutoff value of 11.09 ng/mL (sensitivity, 70.00%; specificity, 95.00%) (Table 1), the serological results were consistent with clinical data (AUC, 0.87; 95% CI, 0.76–0.98) (Figure 5h) with an accuracy of 82.50% (33/40). The above results indicate that KLK12 has good sensitivity and specificity as a biomarker for the serological diagnosis of bovine and human TB and can discriminate between LTBI and active TB. In addition, compared with the TST or Interferon-γ release test, this method also has the advantages of easy operation, fast speed, and high throughput, which makes it easier to popularize for large-scale quarantine work.

## 3. Discussion

MMPs belong to a family of zinc-dependent proteolytic enzymes that can degrade various ECM components. Although most MMPs are expressed in inflamed tissues, some are present in normal tissues, suggestive of their roles in homeostasis [32]. Current evidence suggests that pulmonary epithelial cells can produce many MMPs, including MMP-1, -2, -7, and -9 [33]. In recent years, there has been burgeoning interest in the role of MMPs in pulmonary physiology and pathology. Many MMPs, specifically MMP-1, have been shown to contribute to TB pathology in human lungs [28]. The primary function of MMPs is thought to be matrix cleavage and tissue remodeling. In addition, MMPs can regulate chemokine gradients and leukocyte recruitments to inflammation sites [34]. Accordingly, MMPs play an important role in pulmonary granuloma formation [35,36]. Over the years, MMP inhibitors have been extensively used to study the immunopathology of TB [37,38]. As an adjunctive treatment for TB, the inhibition of MMPs has been shown to significantly reduce granuloma formation and bacterial load [39]. In the present study, MMP-1 and MMP-9 expression levels were upregulated in the lungs of mice infected with *M. bovis* compared to uninfected control mice, consistent, to a certain extent, with previous studies which showed that *Mtb* infection leads to increased expression and activity of MMP-1, -2, -3, and -9 [40]. However, the pathogenesis and regulatory mechanism of MMPs remain poorly understood, emphasizing the need for further research to design appropriate treatment regimens to reduce host damage and improve TB outcomes.

It is well-established that KLKs perform many physiological and pathological functions. The kallikrein–kinin system appears to play a direct role in promoting anti-fibrotic responses and collagen degradation [41]. Given that KLKs can convert kininogens into bradykinin, these kinins then bind to B1R and B2R. Many studies have established that bradykinin stimulation induces the release of MMPs in different tissues. For instance, B1R is reportedly involved in mediating MMP-2 and MMP-9 expression levels [20]. Similarly, B2R has been reported to regulate MMP-9 secretion [21]. These studies suggest that KLKs and MMPs may interact in physiological and pathological conditions such as TB, mediated by BR signaling.

Over the years, several in vitro studies have analyzed the expression of MMPs in the pathophysiology of TB [42]. The expression and secretion of MMPs are strictly regulated, as an excess of these enzymes may cause tissue destruction. Many studies have investigated these signaling pathways. P38/COXII/PGE2/cAMP is a well-established signaling cascade involved in the regulation of MMP-1 [43]. In addition, TNF-α and HDAC1 can also regulate MMP-1 and MMP-9, respectively [28,44,45]. In our present study, the KLK12/BR/ERK signaling pathway could be activated by *M. bovis* or ManLAM, but *MAP* and *M. smegmatis* acted as inhibitors. This may be due to the specific antigen ManLAM on the surface of the *M. bovis* cell wall, whose mannose cap can bind to the cell surface receptors MR, DC-SIGN, and surfactant protein D, which in turn activate downstream signaling pathways [46,47,48]. However, *MAP* and *M. smegmatis* cell walls do not have this specific antigenic component [25]. Exactly which receptor plays the key role remains to be confirmed. In any case, we found that KLK12 regulates the expression of MMP-1 and MMP-9 through the BR/ERK signaling pathway in *M. bovis* infection, which provides a basis for future therapeutic and immunopathological studies of bTB and TB.

In clinical specimens, the differential diagnosis of *Mtb* and NTMs is a significant challenge and often misleading since both *Mtb* and NTMs show positivity in the conventional smear acid-fast staining method. It is likely that a significant proportion of TB patients without a bacteriological diagnosis are actually infected with NTMs or co-infected with NTMs, but the differential diagnosis of TB and NTM disease in this population is not possible due to the limited differential ability of current diagnostic techniques [49]. Therefore, the development of new diagnostic markers and appropriate detection techniques to improve the diagnosis of TB and achieve differential diagnosis with NTM diseases is one of the breakthrough points to improve the efficiency of TB prevention and control. In this study, we substantiated that KLK12 activation is caused by ManLAM, which is only found in pathogenic species such as *M. bovis*, *Mtb*, and *Mycobacterium leprae* [50]. This is due to the special structure of ManLAM mannose caps, which can be recognized by MR, DC-SIGN, and surfactant protein D [46,47,48]. However, the exact receptor that plays a role in the activation of the KLK12/BR/ERK/MMPs signaling pathway needs to be further confirmed. It is well-recognized that ManLAM is a molecule crucial for the ability of *Mtb* to invade and infect host cells [51]. Meanwhile, ManLAM is an especially attractive target for new capture and detection agents [52]. The above findings suggest that KLK12 has considerable diagnostic value as a diagnostic marker for TB upregulation in ManLAM-specific stimulation. Moreover, we collected clinical samples from bTB-positive and bTB-negative cattle diagnosed by the TST. Interestingly, we found that the ManLAM-specific KLK12 response was significantly different between the sera of bTB-positive and bTB-negative cattle, indicating its potential to serve as an immunodiagnostic biomarker of *M. bovis* infection. However, it should be borne in mind that KLK12 mRNA levels are upregulated in cancer tissues, including gastric, breast, and prostate cancer [15]. These types of cancer may affect the clinical diagnosis of TB and affect humans more than cattle, given their susceptibility to these diseases. These findings are consistent with our hypothesis that KLK12 is less effective in diagnosing TB than in diagnosing bTB (Table 1). Importantly, cancer and LTBI were not ruled out in the healthy control subjects in our study and may account for the difference in the diagnostic accuracy between TB and bTB with KLK12.

It has been reported that ~2 billion people in the world harbor LTBI and are at risk of developing active TB disease during their lifetime [27]. However, neither TST nor IFN-γ release assays (IGRAs) can discriminate between active TB and LTBI [53]. Accordingly, developing a rapid diagnostic test to distinguish between active TB and LTBI is paramount for effective TB control. However, given the lack of corresponding animal models, research on the mechanism and diagnosis of LTBI progression to active TB is limited. Herein, mouse models that can simulate different stages of TB were constructed by changing the infection dose. The mouse models provide a new basis for studying LTBI and active TB infection. Moreover, the function of KLK12 and its application in diagnosis were explored in the present study. To the best of our knowledge, this is the first study to report that KLK12 can regulate MMP-1 and MMP-9 expression via bradykinin receptor signaling in *M. bovis*-infected macrophages. The activation of this signaling pathway is caused by the specific antigen ManLAM of *M. bovis* and *Mtb*. Therefore, these cytokines can be used as specific serological biomarkers for the diagnosis of bTB or TB. The value of KLK12 was validated for discriminating between active TB and LTBI or between LTBI and HC. Importantly, the present research provides new animal models and diagnostic methods for TB. With the emergence of drug-resistant *Mtb* [54], our research on the regulatory mechanism of KLK12 may provide reliable targets for improving the effectiveness of existing anti-TB drugs in the future.

## 4. Materials and Methods

### 4.1. Cell Culture

RAW264.7 macrophages were supplied by the Cell Culture Center, Xiehe Medical University (Beijing, China). Macrophages were cultured in a humidified incubator at 37 °C with 5% CO_2_ in DMEM (Hyclone, Logan, UT, USA) supplemented with 10% FBS (Gibco, Grand Island, NY, USA), 100 U/mL penicillin, and 100 µg/mL streptomycin (Gibco). Macrophages were inoculated and grown to 80% confluency prior to transfection or infection.

### 4.2. Bacterial Culture

The virulent *M. bovis* strain C68004 obtained from the China Institute of Veterinary Drug Control has been used for years due to its stable virulence. It was cultured in 7H9 Middlebrook medium (Difco) supplemented with 10% *v*/*v* OADC enrichment solution (BD Biosciences), 2 g/L sodium pyruvate, and 0.05% Tween-80 and incubated at 37 °C in biosafety level 3 (BSL3) facilities, China Agricultural University, Beijing.

### 4.3. Extraction Method of ManLAM

(1) Inactivate *M. bovis* after 4 weeks of culture in a water bath at 65 °C for 2 h, centrifuge at 10,000× *g* for 15 min, collect the organisms, add a small amount of PBS to resuspend cells, and centrifuge again to collect the organisms. (2) Disperse and freeze-dry the inactivated *M. bovis*, resuspend the dried organisms using a solvent mixture (chloroform/methanol, *v*/*v*, 1:1) with 10 times the volume of the organisms, and defat in a shaker at 37 °C for 12 h with vigorous shaking. (3) Centrifuge at 10,000× *g* for 15 min, discard the supernatant to collect the bacterial precipitate, resuspend the organism again in a solvent mixture (chloroform/methanol/water, *v*/*v*/*v*, 10:10:3) with 10 times the volume of the organism, and delipidate by vigorous shaking for 12 h at 37 °C in a shaker. (4) Centrifuge at 10,000× *g* for 15 min, discard the supernatant, collect the defatted organisms, freeze at −80 °C, and then lyophilize. (5) Resuspend the lyophilized bacterium in 5 times the volume of PBS, add DNase containing 200 U/mL, RNase containing 200 U/mL, and PMSF containing 3 mM, mix thoroughly, and measure the volume accurately; then, add Triton X-114 (Solarbio, Beijing, China) to a final concentration of 8% (Triton X-114/PBS, *v*/*v*), mix thoroughly again, and leave for 12 h at 4 °C. (6) Centrifuge at 32,000× *g* for 30 min at low temperature, carefully aspirate the upper liquid phase in a sterile tube, and place in a 37 °C incubator until clearly stratified. (7) Take the lower Triton X-114 liquid phase, add 9 times the volume (of Triton X-114 phase) of 95% ice ethanol, and place it at −80 °C for 12 h until the precipitate forms. (8) Centrifuge at 10,000× *g* for 5 min at low temperature, discard the supernatant to collect the precipitate, lyophilize, and then add 2 times the volume of a final concentration of 2 mg/mL proteinase K solution in a water bath at 58 °C for 2 h. (9) After dialysis, to remove small molecules such as amino acids, collect the liquid in the dialysis bag and lyophilize again. (10) Add the extracts to the loading buffer and subject them to SDS-PAGE electrophoresis. After electrophoresis, excise the concentrated gel and stain the whole isolated gel with the glycogen PAS staining kit (Solarbio, Beijing, China).

### 4.4. siRNA Transfection

According to our previous research, 20 nmol/L small interfering RNA targeting KLK12 and negative siRNA (Synbio Technology, Beijing, China) were used to transfect RAW264.7 macrophages using Lipofectamine 3000 (Invitrogen, Carlsbad, CA, USA). The sequences of the siRNAs used in the current study were as follows: KLK12 siRNA sense, 5′-UCAGAACCAUGAGCAUGAUTT-3′; KLK12 siRNA antisense, 5′-AUCAUGCUCAUGGUUCUGATT-3′; negative siRNA sense, 5′-UUCUCCGAACGUGUCACGUTT-3′; negative siRNA antisense, 5′-ACGUGACACGUUCGGAGAATT-3′. After 6 h of transfection, the medium was replaced with fresh medium supplemented with 10% FBS. After 48 h post-transfection, KLK12 siRNA- and negative control siRNA-treated macrophages were infected with *M. bovis* C68004.

### 4.5. Bacterial Infection

RAW264.7 macrophages were infected with *M. bovis* C68004 at a multiplicity of infection (MOI) of 10. After 3 h of incubation, fresh DMEM medium supplemented with 2% FBS was added, and macrophages were incubated for indicated periods. 

Six–eight-week-old C57BL/6 female mice (Vital River Laboratories, Beijing, China) were distributed into three groups (*n* = 20). The conventional-dose (CD) infection group was inoculated intranasally with *M. bovis* C68004 at a dose of 200 CFU/mice, the ultra-low-dose (ULD) infection group was inoculated with *M. bovis* C68004 at a dose of 2 CFU/mice, and the negative group was inoculated intranasally with the same volume of phosphate-buffered saline (PBS). 

### 4.6. Western Blot

For Western blot analysis, samples were obtained from cultured macrophage cells lysed using RIPA buffer with phosphatase and protease inhibitors (Solarbio, Beijing, China). The samples were placed on ice for 20 min, and the SDS loading buffer was added. The homogenized samples were boiled for 10 min. Later on, the different proteins were analyzed according to standard Western blot procedures. The primary antibodies were: Rabbit polyclonal anti-KLK12 antibody (BioVision Incorporated, Milpitas, CA, USA), Rabbit polyclonal MMP1 antibody, Rabbit polyclonal MMP9 antibody, and Rabbit polyclonal GAPDH antibody (Proteintech, Wuhan, Hubei, China).

### 4.7. Quantitative Real-Time PCR

Quantitative Real-Time PCR (qRT-PCR) was used to study the effect of different infection doses on the expression of KLK12, MMP-1, and MMP-9 in mouse lung tissue. Total RNA was isolated using the RNA extraction kit (Aidlab Biotechnology, Beijing, China). The concentration and purity of the yielded RNA were measured using NanoDrop 2000. RNA samples were converted to cDNA using the RevertAid First Strand cDNA synthesis kit (Thermo Scientific, Massachusetts, USA). We amplified the mRNAs of various genes using the ACEQ qPCR SYBR Green Master Mix kit (Vazyme Biotech, Nanjing, China). All primers used in the current study are displayed in Table 2. The fold changes in the relative expression levels of various genes were obtained by the 2^−ΔΔCt^ protocol.

### 4.8. ELISA

Serum levels of various cytokines were measured using ELISA kits according to the manufacturer’s instructions. The cytokines tested included IFN-γ, TNF-α, and IL-17 (Neobioscience Technology, Shenzhen, China) and KLK12, MMP-1, and MMP-9 (Jonln, Shanghai, China).

### 4.9. CFU Assay

To count the number of colonies of *M. bovis*, cells or tissue suspensions were lysed with 0.1% Triton X-100. The lysates were diluted with PBS and plated in triplicate on Middlebrook 7H10 agar plates supplemented with polymyxin, amphotericin B, sodium pyruvate, and OADC. Inoculated plates were incubated at 37 °C, and colonies were counted after three to four weeks.

### 4.10. Statistical Analysis

The data from cell experiments were expressed as the mean ± SD of three independent experiments. For comparison between two groups, Student’s t-test was applied, and one-way ANOVA was used for comparisons of more than two groups. ImageJ software (National Institute of Health, Bethesda, MD, USA) was used for densitometric analysis of Western blot images. A *p*-value less than 0.05 was statistically significant.

## 5. Patents

There is a patent resulting from the work reported in this manuscript. The patent name is “Serological Diagnostic Markers for Bovine Tuberculosis and its Clinical Application”. The application (patent) number is “CN202111208878.0”.

## Figures and Tables

**Table 1:** Determination of the cutoff values for bTB and TB diagnosis.

Role	AUC ^1^	Sensitivity (%)	95% CI ^2^ (%)	Specificity (%)	95% CI (%)	Cutoff Value (ng/mL)
Identification of LTBI and healthy cattle	0.92	81.93	72.30–88.73	80.00	49.02–96.45	>16.49
Identification of active bTB and LTBI cattle	0.94	100.00	81.57–100.00	85.54	76.41–91.53	>41.55
Identification of active TB and healthy humans	0.87	70.00	48.10–85.45	95.00	76.39–99.74	>11.09

^1^ Area under the curve; ^2^ 95% confidence interval.

**Table 2:** Primers used in the present study.

Gene Name	Forward Primers (5′-3′)	Reverse Primers (5′-3′)
*GAPDH*	TGCACCACCAACTGCTTAG	GGATGCAGGGATGATGTTC
*KLK12*	CAGCCAGACTCTCTGGTTCC	TCCAGCCCCTAGCTAACAGA
*MMP-1*	TTCCCCAAATCCCATCCAGC	ACCCGAATGTAGAACCTGCC
*MMP-9*	TACTGGGCGTTAGGGACAGA	TAACGCACAGACCCCCTCTA
*B1R*	AGCACCAGCTGCTCATCTACC	GACCGGCGAAAGAGGATATAGAC
*B2R*	CAGCACCTTCCTGGATACGCTGCATC	CACCTCCCAAGACTTCTTTCGGAAGC

## Data Availability

Not applicable.

## Acronyms

BR	Bradykinin receptors
MOI	Multiplicity of infection
POC	Point-of-care
ULD	Ultra-low dose
CD	Conventional dose
H&E	Hematoxylin and eosin
NC	Negative control
HC	Healthy control
ROC	Receiver operating characteristic
AUC	Area under the curve
CI	Confidence interval
FMD	Foot and mouth disease
TST	Tuberculin skin test
